# Effect of filter extraction solvents on the measurement of the oxidative potential of airborne PM_2.5_

**DOI:** 10.1007/s11356-021-12604-7

**Published:** 2021-02-09

**Authors:** Maria Chiara Pietrogrande, Dimitri Bacco, Arianna Trentini, Mara Russo

**Affiliations:** 1 Department of Chemical and Pharmaceutical Sciences, University of Ferrara, Via Fossato di Mortara 17/19 - 44121, Ferrara, Italy; 2 Emilia Romagna Regional Agency for Prevention, Environment and Energy, ARPAE, Via Po 5 - 40139, Bologna, Italy

**Keywords:** PM_2.5_, Oxidative potential, Filter extraction solvents, Phosphate buffer, Gamble’s solution, Methanol, DTT and AA assays

## Abstract

Solvent extraction of PM_2.5_ samples collected on the filter is a preliminary step for assessing the PM_2.5_ oxidative potential (OP) using cell-free assays, as the dithiothreitol (DTT) and the ascorbic acid (AA) assays. In this study, we evaluated the effect of the solvent choice by extracting ambient PM_2.5_ samples with different solvents: methanol, as organic solvent, and two aqueous buffers, i.e., phosphate buffer (PB) and Gamble’s solution (G), as a lung fluid surrogate solution. Both the measured volume-based OP_V_^DTT^ and OP_V_^AA^ responses varied for the different extraction methods, since methanol extraction generated the lowest values and phosphate buffer the highest. Although all the tested solvents produced intercorrelated OP_V_^DTT^ values, the phosphate buffer resulted the most useful for OP^DTT^ assessment, as it provided the most sensible measure (nearly double values) compared with other extractions. The association of the measured OP_V_ values with PM chemical composition suggested that oxidative properties of the investigated PM_2.5_ samples depend on both transition metals and quinones, as also supported by additional experimental measurements on standard solutions of redox-active species.

**Supplementary Information:**

The online version contains supplementary material available at 10.1007/s11356-021-12604-7.

## Introduction

Many epidemiological and toxicological studies have demonstrated that oxidative stress is one of the main mechanisms by which atmospheric particles (PM) can trigger negative health effects , mediated by the generation and subsequent reactions of reactive oxygen species (ROS) and reactive nitrogen species (RNS) (Akhtar et al. 2010; Bates et al. 2015; Longhin et al. 2013; Verma et al. 2015). PM oxidative potential (OP) has been thus defined as a measure of the ability of PM components to oxidize a target molecule or to catalyze the production of ROS/RNS and consumption of antioxidants. Thus, it could represent a better metric of PM hazard exposure than PM mass concentration, as an unifying metric integrating PM chemical/physical properties and bioreactivity into one measurement (Abrams et al. 2017; Antinolo et al. 2015; Calas et al. 2017; Crobeddu et al. 2020; Fang et al. 2016; Janssen et al. 2015; Jiang et al. 2019; Øvrevik 2019; Romano et al. 2019). Among several in vitro cellular and acellular assays proposed to quantify OP, cell-free assays, besides being non-invasive, have the advantages of being fast, inexpensive, easy to organize and suitable for automation (Bates et al. 2019; Calas et al. 2018; Fang et al. 2015; Gao et al. 2017; Hedayat et al. 2014; Pietrogrande et al. 2019a; Visentin et al. 2016). They all require a preliminary solvent extraction to retrieve the PM components from the loaded filter, still conserving, as much as possible, the physical and chemical properties of the complex mixtures present in the atmosphere. This is a critic step, as the major contributors to PM toxicity are trace components, that contribute little to PM mass and may be potentially loss during the extraction process or contaminated by interfering compounds (Bein and Wexler 2015; Roper et al. 2017; Roper et al. 2019; Simonetti et al. 2018; Van Winkle et al. 2015). Although several OP assay procedures are currently in use, to date, no real consensus has emerged towards standardized protocols, including the type of filters used to collect atmospheric PM, solvent and extraction procedures, and OP assay media (Bates et al. 2019; Calas et al. 2017; Luo et al. 2019; Molina et al. 2020; Roper et al. 2015; Roper et al. 2017; Wiseman 2015; Yang et al. 2014). This may generate discrepancies in assay responses reducing the reliability of OP data and, hence, lessen formation of a robust consensus on the use of OP as an exposure metric for ambient air PM in epidemiological studies.

This motivates the present study, that investigates the impact of the filter solvent extraction on responses of two acellular OP assays, in order to guide selection of an extraction method that is best suited for OP assessments of ambient PM_2.5_ samples. The study was performed by extracting equal portions of a single PM_2.5_ filter with different solutions, namely, phosphate buffer and methanol, as typically used for PM extraction in OP assays and toxicological studies. In addition, the Gamble’s solution was investigated, as a lung fluid surrogate mixture, that may closely mimic bio-accessibility of particle-bound species in the physiological fluids encountered during PM exposition (Calas et al. 2017; Collins et al. 2015; Leclercq et al. 2017; Luo et al. 2019; Mukhtar et al. 2015; Van Winkle et al. 2015; Wiseman 2015; Zeng et al. 2019). To date, the impact of solvent extraction on PM OP or toxicity has been studied by comparing aqueous solutions vs. organic solvents (i.e., Bein and Wexler 2015; Roper et al. 2019; Yang et al. 2014), or different simulated lung fluid surrogates (i.e., Calas et al. 2017; Leclercq et al. 2017; Van Winkle et al. 2015). To our knowledge, a comprehensive inter-comparison of the three different extraction liquids has never been conducted.

Two widely used cell-free assays were investigated: the dithiothreitol (DTT) assay, which simulates PM-catalyzed electron transfer from cellular antioxidants (e.g., NADPH) to O_2_ and the ascorbic acid (AA) assay, based on redox reactions of AA as the most abundant antioxidant found in lung fluids, which has a vital role based on the catalytic ability of redox-active species (Bates et al. 2019; Calas et al. 2018; Charrier and Anastasio 2015; Crobeddu et al. 2020; Fang et al. 2016; Gao et al. 2017; Hedayat et al. 2014).

The study was performed on 32 real environmental PM_2.5_ samples from urban and rural sites in the Po Valley (Northern Italy) in spring 2018, as well as on individual standard compounds, chosen for either their known reactivity to the DTT and AA assays or their abundance in atmospheric aerosol, i.e, metals and quinones (Charrier and Anastasio 2015; Fang et al. 2016; Fujitani et al. 2017; Pietrogrande et al. 2019a; Verma et al. 2015; Tuet et al. 2017).

The study is a part of a larger project concerning a critical investigation of the different experimental protocols generally used in OP assays, with the aim to provide useful information to design a standardized analytical protocol for increasing the reliability and overall quality of data derived from OP assays (Pietrogrande et al. 2019a; Pietrogrande et al. 2019b).

## Materials and methods

### Reagents and standards

Sodium phosphate (NaH_2_PO_4_, ACS), Magnesium chloride hexahydrate (MgCl_2_^.^6H_2_O), sodium chloride (NaCl, ACS), potassium chloride (KCl), disodium hydrogen phosphate (Na_2_HPO_4_), sodium Sulfate (Na_2_SO_4_), calcium chloride dehydrate (CaCl_2_.2H_2_O), sodium acetate, sodium hydrogen carbonate (NaHCO_3_), and sodium citrate dehydrate (C_6_H_5_O_7_Na_3_·2H_2_O) were from Fisher Scientific.

Standard solutions of L-ascorbic acid sodium salt (AA), (Sigma Aldrich) were prepared at 10 mM concentration in ultrapure water (Milli-Q® IQ 7000 water purification system). Solutions of DTT and DTNB (Sigma Aldrich) were prepared in phosphate buffer (at 10 mM) and maintained in ice and in the dark during the experiment.

Aqueous solutions of the reagents are unstable at room temperature and sensible to light, thus they were preserved in amber glass vials in the dark at − 20 °C.

Copper (II) sulfate (98 %), iron (II) chloride (ACS), 1,2-naphthoquinone (1,2-NPQ, 97 %), and 9,10-phenanthrenequinone (9,10-PNQ, 99%) were from Sigma-Aldrich and Acros Organics. Individual standard stock solutions were prepared for each analyte by weighting pure standards with a concentration of 10^−2^ M using MilliQ water for metal ions and acetonitrile for quinones as solvent.

Methanol was HPLC grade solvent purchased from Sigma-Aldrich.

### Study sites and PM sampling

Sampling took place at two sites in the Emilia Romagna region, in the eastern part of the Po Valley (northern Italy). The urban background site (URB) is located in the middle of the city of Bologna (~ 400,000 inhabitants) in a densely populated area, and the rural background station (RUR) is located at San Pietro Capofiume, about 30 km northeast from the city.

From 10 March to 8 April 2018, four PM_2.5_ samples were simultaneously collected every day at the two sites. A low volume automatic outdoor sampler (Skypost PM, TCR-TECORA Instruments, Corsico, Milan, Italy) was used, operating at the standard airflow rate of 38.3 L min^−1^ for 24 h to collect an air volume of 55 m^3^ per day. PM_2.5_ samples were collected on 47-mm diameter quartz fiber filters provided from Whatman (Whatman® QM-A quartz filters). After sampling, the procedure outlined in European Standard EN 12341 (CEN, 1998) was applied for equilibration and weighing the collected samples. All details concerning the site and the logistical aspects of the sampling procedure can be found in Authors’ papers (Pietrogrande et al. 2019a; Ricciardelli et al. 2017; Visentin et al.^ 2016^).

### Extraction solvent and solutions

The phosphate buffer (Na_2_HPO_4_ and NaH_2_PO_4_) was 0.1 M at pH 7.4. The Gamble’s solution was prepared with magnesium chloride hexahydrate 10^−3^ M, sodium chloride 0.1 M, Potassium chloride 4^.^10^−3^ M, disodium hydrogen phosphate 0.9^.^10^−3^ M, sodium sulfate 0.4^.^10^−3^ M, calcium chloride dehydrate 2.5^.^10^−3^ M, sodium acetate 7^.^10^−3^ M, sodium hydrogen carbonate 0.03 M, and sodium citrate dehydrate 0.3^.^10^−3^ M in ultrapure water.

All solutions were prepared in ultrapure water (Milli-Q® IQ 7000 water purification system). Both phosphate buffer and Gamble’s solution were treated with Chelex® 100 sodium form resin (BioRad) to remove any metal contamination.

### Extraction procedures

In our experiments, each quartz filter was divided into four parts, and three of them were extracted using each of the investigated solvents—phosphate buffer, Gamble’s solution, and methanol—and then submitted to DTT and AA assays. The same procedure was also applied to quartz blank filters spiked with standard solutions of redox active compounds and then extracted with the 3 solvents and also with ultrapure Milli-Q water.

#### Aqueous solution extraction

Water-based extractions were performed for 15 min in an ultrasonic bath using 10 mL of phosphate buffer or Gamble’s solution, following the procedure previously used by Authors that has been found to guarantee a good extraction efficiency ≥ 90% (Pietrogrande et al. 2019a; Visentin et al. 2016). The extracts were then filtered on a regenerate cellulose syringe filter (13 mm, 0.22 μm, Kinesis) to remove the suspended solid particles. Then, 3 mL of the solution were submitted to each OP assays.

#### Methanol extraction

A quarter of filter was extracted for 15 min in an ultrasonic bath using 10 mL of methanol, following the procedure commonly used by other Authors (Bein and Wexler 2015; Gao et al. 2017; Janssen et al. 2015; Roper et al. 2017; Roper et al. 2019; Verma et al. 2015; Yang et al. 2014). The extract was then filtered on a PTFE syringe filter (25 mm, 0.22 μm, Kinesis) to remove the suspended solid particles. The filtrate was then transferred into a rounded glass flask and placed in a centrifugal vacuum concentrator (miVac, Genevac Inc, USA) to remove methanol to dryness. Then, extract was reconstituted by adding 10 mL of phosphate buffer, and 3 mL of the solution were submitted to each OP assay.

### DTT and AA assays for measuring oxidative potential

Oxidative potential of the collected PM_2.5_ samples and standard solutions were assessed with the DTT and AA assays, following the experimental procedure described elsewhere (Perrone et al. 2019; Pietrogrande et al. 2018; Visentin et al. 2016). Briefly, both the assays were performed on 3 mL of the filter extract operating at a constant temperature of 37 °C using a dry bath.

Spectrophotometric measurements were performed in a UV-Vis spectrophotometer (Jasco V-730, JASCO EUROPE s.r.l.) with a 1-cm path length optical cell. Polystyrene and quartz cuvette were used for DTT and AA assays, respectively.

In the DTT assay, 30 μl of the 10 mM DTT solution was added to the sample (i.e., time zero) and the rate of DTT depletion (OP^DTT^) measured as follows. At defined times, a 0.50 mL aliquot of the reaction mixture was removed, and the reaction stopped with trichloroacetic acid (0.50 mL of 10%). Then, the remaining DTT was reacted with DTNB (5,5′-Dithiobis(2-nitrobenzoic acid)) to generate DTT-disulphide and 2-nitro-5-thiobenzoic acid (TNB): 50 μL of the DTNB solution (10 mM concentration in phosphate buffer at pH 7.4) was added to each aliquots and well mixed. After 2 min to allow the complete reaction, pH was increased to pH 8.9 by adding 2.0 mL of Tris-HCl buffer (0.40 M at pH 8.9 with 20 mM of EDTA) to form the mercaptide ion (TNB^2−^), which has a high absorbance (molar extinction coefficient *ε* = 14150 M^−1^ cm^−1^ at 412 nm).

In the AA assay, 30 μl of the 10 mM AA solution was added to the sample (i.e., time zero). Then, the rate of AA depletion (OP^AA^) was followed directly in the spectrophotometric cuvette by measuring at defined time intervals the absorption of the ascorbate ion at 265 nm (*ε* = 14500 M^−1^ cm^−1^ at pH 7.4).

The rate of DTT or AA depletion (nmol min^−1^) was determined by linearly fitting the experimental points of the reagents concentration versus time (5, 10, 15, 25, 40 min). The response of blank filters was determined and subtracted from the data of real PM samples. The obtained OP responses were then normalized both to air collected volume, i.e., volume-normalized OP_V_ (nmol min^−1^ m^−3^), and to PM_2.5_ sampled mass, i.e., mass-normalized OP_m_ (nmol min^−1^ μg^−1^).

### Chemical analysis of ambient PM_2.5_ samples

Chemical composition of the PM_2.5_ samples was investigated, by quantifying the main tracers useful for source apportionment. Chemical analysis was performed in the laboratories of the Emilia Romagna Regional Agency for Prevention, Environment, and Energy in Ravenna (Italy), using 3 PM_2.5_ samples simultaneously collected each day. Details are reported elsewhere (Ricciardelli et al. 2017).

Briefly, one PM_2.5_ filter was extracted in 10 ml of MilliQ water, sonicated for 15 min, filtered on 0.45-μm cellulose acetate filter and then submitted to the following instrumental analyses. Inorganic ions were quantified by Ionic Chromatography: ICS-1000 with IonPac™AS9-HC for anions (Cl^−^, NO_3_^−^, SO_4_^2−^) and ICS-1100 with IonPac™CS12A for cations (K^+^, NH_4_^+^) (DIONEX, California, USA). Levoglucosan was quantified using HPLC-MS instrument (HPLC Agilent 1200 series and Triple Quadrupole 6410 equipped with Electrospray Ionization, Agilent Technologies Inc., CA, USA) with a ZORBAX amino column. One filter was mineralized with 10 ml of a HNO_3_:H_2_O_2_ (8:2) mixture and analyzed for metal quantification using inductively coupled plasma–mass spectrometry (7700 ICP-MS, Agilent Technologies Inc., CA, USA), following the method reported in UNI EN 14902:2005. Another filter was directly submitted to thermo-optical transmission analysis to quantify the carbonaceous fraction, i.e., elemental carbon, EC, and organic carbon, OC. A Sunset instrument (Laboratory Inc., OR, USA) was used, following the EUSAAR2 thermal protocol (Cavalli et al. 2010), according to the European standard (UNI EN 16909:2017).

### Statistical analysis

The paired sample *t* test was used to examine whether extraction solvent differed significantly for each OP assay, as well as samples collected at the urban and rural sites. A *p* value less than 0.05 was regarded as statistically significant.

Pearson correlation analysis was performed to investigate significance of correlations among OP responses for different extraction methods as well as associations between OP responses and chemical composition.

## Results and discussion

Three extraction solvents were investigated to represent the typically used protocols for OP assay of PM samples:

- phosphate buffer (PB), the most common aqueous buffer employed by the Authors (Perrone et al. 2019; Pietrogrande et al. 2018; Pietrogrande et al. 2019a; Visentin et al. 2016) and other researchers (Bates et al. 2019; Calas et al. 2018; Fang et al. 2015; Hedayat et al. 2014);
- methanol (Me), the organic solvent frequently used in PM_2.5_ toxicology studies*,* due to its ability to extract hydrophobic and hydrophilic compounds, combined with its low cost and comparatively small blank filter effect (Bein and Wexler 2015; Janssen et al. 2015; Roper et al. 2017; Roper et al. 2019; Verma et al. 2015; Yang et al. 2014);
- Gamble’s solution (G), an artificial lung fluid consisting of a mixture of salts (pH: 7.4) widely used for closely simulating the real physiological conditions related to PM exposition. It is typically employed for pulmonary simulation in studies on PM toxicity, since it represents the extra-cellular fluids in the deep lung (Bein and Wexler 2015; Collins et al. 2015; Goix et al. 2016; Leclercq et al. 2017; Mukhtar et al. 2015; Wei et al. 2019; Wiseman, 2015; Xing et al. 2019; Zeng et al. 2019).

Toxicological studies suggest that biological response of particle-bound species is strongly affected by different extraction protocols, since it is strongly influenced by the degree of bio accessibility of the biologically active compounds, i.e., immediately internalized compounds can cause faster inflammation than insoluble species in lung fluids (Roper et al. 2017; Xing et al. 2019).

The different extraction methods were tested by measuring OP^DTT^ and OP^AA^ responses of each ambient PM_2.5_ filter after extraction with the three liquids and comparing the obtained results.

### OP^DTT^ and OP^AA^ responses for ambient PM samples in the three extracting solutions

**Fig. 1:**
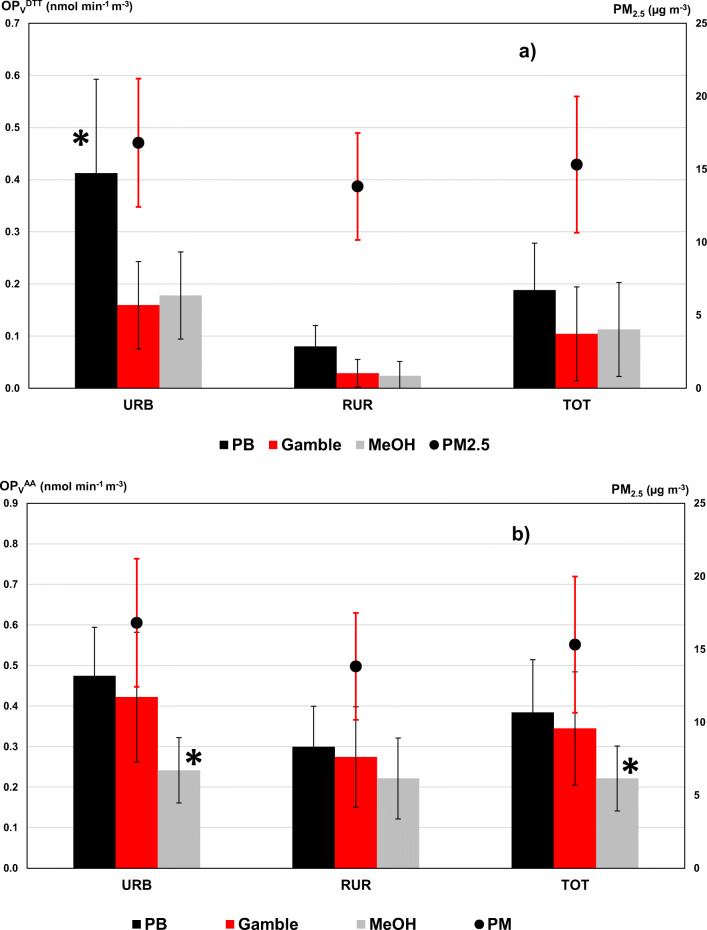
Comparison among OP_V_ responses using different extraction solvents. Mean values were computed on all the investigated PM_2.5_ samples, as well as on urban and rural samples, separately. Error bars denote 1 standard deviation. ^*^indicates statistical significance at *p* < 0.05 level. Black bars: phosphate buffer; red bars: Gamble’s solution; light grey bars: methanol. **a**. Comparison among OP_V_^DTT^ responses. **b** Comparison among OP_V_^AA^ responses.

The individual volume- and mass normalized OP^DTT^ and OP^AA^ responses were measured for each sample (Tables S1-S3 in Supplementary Material), and their mean values were computed for each investigated solvent (Table 1). The mean OP_V_^DTT^ and OP_V_^AA^ responses are compared in Fig. 1a, b (light grey bars). Overall, similar OP values were measured from the different extracts, with mean OP_V_^DTT^ values ranging from 0.10 ± 0.09 to 0.19 ± 0.18 nmol min^−1^ m^−3^ and mean OP_V_^AA^ from 0.22 ± 0.08 to 0.38 ± 0.16 nmol min^−1^ m^−3^. In general, such measured activities are at the lower end of the range usually measured for PM_2.5_ at Italian sites, i.e., ~ 0.2–2.0 nmol min^−1^ m^−3^, with low values typical for spring/summer (Perrone et al. 2019; Pietrogrande et al. 2018; Pietrogrande et al. 2019a, b; Simonetti et al. 2018; Visentin et al. 2016).

**Table 1:** Experimental parameters measured in PM_2.5_ particles: mean values and standard deviation (SD) computed for all the investigated samples (total, *n* = 32) and the samples collected at the urban (*n* = 16) and rural sites (*n* = 16), separately. OP^DTT^ and OP^AA^ responses were measured after extraction with each investigated solvent and expressed as volume-based OP_V_ (nmol min^−1^ m^−3^) and mass-based OP_m_ (nmol min^−1^ μg^−1^) values. Concentrations of chemical components are expressed in ng m^−3^, unless differently specified. ^*^Indicates statistically significant difference (*p* < 0.05) among the extraction solvents; ^**†**^indicates statistically significant difference between urban and rural samples

	Total (n = 32)		Urban(n = 16)		Rural (n = 16)	
	Mean	SD	Mean	SD	Mean	SD
OP_V_^DTT^ PB (nmol min^−1^ m^−3^)	0.19	0.18	0.41*	0.18	0.08**†**	0.04
OP_V_^DTT^ G (nmol min^−1^ m^−3^)	0.10	0.09	0.16	0.08	0.03**†**	0.03
OP_V_^DTT^ MeOH (nmol min^−1^ m^−3^)	0.11	0.09	0.18	0.08	0.02**†**	0.03
OP_V_^AA^ PB (nmol min^−1^ m^−3^)	0.38	0.16	0.47	0.15	0.30	0.12
OP_V_^AA^ G (nmol min^−1^ m^−3^)	0.34	0.22	0.42	0.26	0.27	0.12
OP_V_^AA^ MeOH (nmol min^-1^ m^-3^)	0.22*	0.08	0.24*	0.08	0.22	0.14
OP_m_^DTT^ PB (nmol min^−1^ μg^−1^)	0.014	0.008	0.023	0.018	0.002**†**	0.002
OP_m_^DTT^ G (nmol min^−1^ μg^−1^)	0.006	0.004	0.009	0.003	0.002**†**	0.002
OP_m_^DTT^ MeOH (nmol min^−1^ μg^−1^)	0.006	0.005	0.010	0.006	0.001**†**	0.001
OP_m_^AA^ PB (nmol min^−1^ μg^−1^)	0.027	0.015	0.031	0.016	0.024	0.013
OP_m_^AA^ Gamble (nmol min^-1^ μg^-1^)	0.023	0.013	0.026	0.013	0.021	0.013
OP_m_^AA^ MeOH (nmol min^−1^ μg^−1^)	0.013	0.012	0.012	0.009	0.016	0.015
PM_2.5_ (μg m^−3^)	15.32	4.67	16.81	5.19	13.83**†**	3.66
OC (μg m^−3^)	3.95	1.41	4.42	1.49	3.48	1.14
EC (μg m^−3^)	0.79	0.41	1.04	0.43	0.54**†**	0.17
Levoglucosano	117.81	60.69	135.18	52.75	100.44	63.10
NH_4_^+^ (μg m^−3^)	1.83	0.72	1.93	0.77	1.74	0.66
K^+^	84.69	43.87	92.50	42.20	76.88	44.12
Cl^-^	70.63	88.14	141.25	74.57	< LOD**†**	-
NO_3_^-^ (μg m^−3^)	3.43	1.65	3.74	1.76	3.12	1.46
SO_4_^2-^ (μg m^−3^)	1.54	0.81	1.65	0.81	1.42	0.80
Metals	91.89	144.16	166.46	82.56	26.65**†**	16.14
Fe	80.84	123.03	128	58.38	33.68**†**	27.24
Mn	1.89	1.32	2.49	1.63	1.33	0.48
Zn	0.46	0.69	< LOD	-	0.92	0.73
Pb	4.58	5.60	10.21	3.52	< LOD**†**	-
V	1.89	1.23	2.33	1.48	1.47**†**	0.69

A deeper insight into the data highlights that the OP responses varied for different extraction methods, since methanol extraction generated the lowest values and phosphate buffer the highest, following the same increasing order for both OP_V_^DTT^ and OP_V_^AA^ responses:

methanol < Gamble < phosphate buffer

However, given the large standard deviations of the calculated means, statistically significant difference among the solvent data (Student’s *t* test, *p* < 0.05) was singled out only for lower methanol OP_V_^AA^ data (signified by * in Fig. 1b, light grey bars). Indeed, such SDs do not reflect uncertainties in sample analysis (% RSD ≤ 6% for all measurements), but rather variations in the OP responses of the real investigated PM_2.5_ filters.

### Comparison between urban and rural PM samples

Then, data from filters collected at the urban (URB, *n* = 16) and rural (RUR, *n* = 16) sites were separated and investigated in detail. The objective was not to compare the different locations, but rather to explore the effect of solvent extraction on OP of PM samples with different chemical compositions. The mean and SD values of volume-based OP^DTT^ and OP^AA^ responses computed on the two groups are reported in (Table 1) and shown in Fig. 1a, b for comparison.

The two separated groups showed the same general solvent extraction trend, even with magnified differences for the URB compared with RUR samples. In fact, for URB samples, OP_V_^DTT^ responses with PB were nearly double that those with the other solvents (significant differences indicated by * in Fig. 1a). Otherwise, the RUR group showed almost constant OP_V_^DTT^ and OP_V_^AA^ data, nearly independent of the extraction conditions (Tables S1 and S2 in Supplementary Material). By comparing the OP_V_^DTT^ and OP_V_^AA^ values measured at the two sites in detail, we can observe different behaviors of the two OP assays.

Overall, the OP_V_^DTT^ values measured in Bologna are significantly higher (*p* < 0.05, **†** in Table 1) than those at the rural site, for each investigated solvent. Such differences may be mainly ascribed to variations in PM_2.5_ mass concentration, that follow the same trend with higher mean values at URB than at RUR sites, i.e., 16.8 ± 5.2 μg m^-3^ and 13.8 ± 3.7 μg m^-3^, respectively (Table 1 and Fig. 1a, b).

In addition, they may be generated from differences in the PM intrinsic oxidative properties, quantified by mass-related OP_m_^DTT^ parameter (Table 1 and S3). In fact, these values are significantly (*p* < 0.05) higher at URB (from 0.009 ± 0.003 to 0.023 ± 0.018 nmol min^−1^ μg^−1^) compared with RUR site (from 0.001 ± 0.001 to 0.002 ± 0.002 nmol min^−1^ μg^−1^) (Table S3). Such a difference may be ascribed to likely higher concentrations of redox-active PM components at the urban site (Pietrogrande et al. 2018; Pietrogrande et al. 2019a, b; Simonetti et al. 2018; Visentin et al. 2016).

On the contrary, the measured OP^AA^ responses, both volume- and mass-related values, do not show any significant difference between the two sites (Tables S1-S3). This is consistent with several literature papers reporting that the AA assay reactivity is less dependent on PM mass concentration as well as on chemical composition than the DTT response (Bates et al. 2019; Calas et al. 2018; Fang et al. 2016; Hedayat et al. 2014; Janssen et al. 2015; Perrone et al. 2019; Pietrogrande et al. 2019a; Visentin et al. 2016).

It is noteworthy that such difference/similarity pattern between the sites is the same for the three extraction protocols, suggesting the independence of the solvent type.

### Correlations between OP responses: effect of the extracting solutions and of different assays

To highlight the effect of the extraction solvent on OP responses of different samples, the intercorrelation between OP data measured after each extraction procedure was explored by Pearson correlation analysis on the whole dataset (Table 2) and by separately investigating URB and RUR samples (Tables S4 and S5).

**Table 2:** Pearson inter-correlation coefficients of OP_V_ (nmol min^−1^ m^−3^) responses with concentration of PM_2.5_ mass and chemical components for all PM_2.5_ samples. OP_V_ were measured after extraction with phosphate buffer, Gamble’s solution, and methanol. Concentrations of chemical components are expressed in ng m^−3^, unless differently specified. Significant *r* values based on a two-tailed *t* test (*n* = 32) are reported in **bold (**at ***p*** **level < 0.01**) and in *italic (at p level < 0.05*)

	OP_V_^DTT^ PB	OP_V_^DTT^ G	OP_V_^DTT^ MeOH	OP_V_^AA^ PB	OP_V_^AA^ G	OP_V_^AA^ MeOH
OP_V_^DTT^ PB (nmol min^-1^ m^-3^)	1.00					
OP_V_^DTT^ G (nmol min^-1^ m^-3^)	**0.683**	1.00				
OP_V_^DTT^ MeOH (nmol min^-1^ m^-3^)	**0.945**	**0.807**	1.00			
OP_V_^AA^ PB (nmol min^-1^ m^-3^)	0.175	*0.382*	0.226	1.00		
OP_V_^AA^ G (nmol min^-1^ m^-3^)	0.162	*0.387*	0.255	**0.605**	1.00	
OP_V_^AA^ MeOH (nmol min^-1^ m^-3^)	0.113	0.148	0.155	**0.456**	*0.431*	1.00
PM_2.5_ (μg m^-3^)	**0.541**	**0.770**	**0.651**	0.253	0.305	0.075
OC (μg m^-3^)	*0.423*	**0.674**	**0.554**	0.227	*0.390*	-0.045
EC (μg m^-3^)	**0.507**	**0.781**	**0.631**	**0.486**	**0.532**	0.161
Levoglucosan	0.359	*0.376*	0.427	0.161	*0.430*	-0.118
NH_4_^+^ (μg m^-3^)	0.307	*0.361*	0.371	-0.136	0.059	-0.051
K^+^	0.319	*0.419*	0.421	-0.012	*0.387*	0.011
Cl^-^	**0.471**	*0.402*	**0.472**	**0.454**	0.324	-0.137
NO_3_^-^ (μg m^-3^)	0.240	*0.360*	0.305	0.002	0.045	-0.065
SO_4_^2-^ (μg m^-3^)	**0.452**	*0.379*	**0.496**	-0.186	0.076	0.114
Total Metals	0.239	**0.643**	*0.399*	0.114	0.156	0.245
Fe	0.335	**0.539**	*0.423*	0.280	0.222	0.296
Mn	0.363	**0.609**	**0.473**	0.293	0.290	*0.442*
Zn	0.083	*0.442*	0.232	*0.428*	*0.420*	0.264
Pb	0.268	**0.871**	**0.755**	**0.498**	**0.480**	0.292
V	*0.432*	**0.473**	**0.675**	-0.033	0.025	0.050

**Fig. 2:**
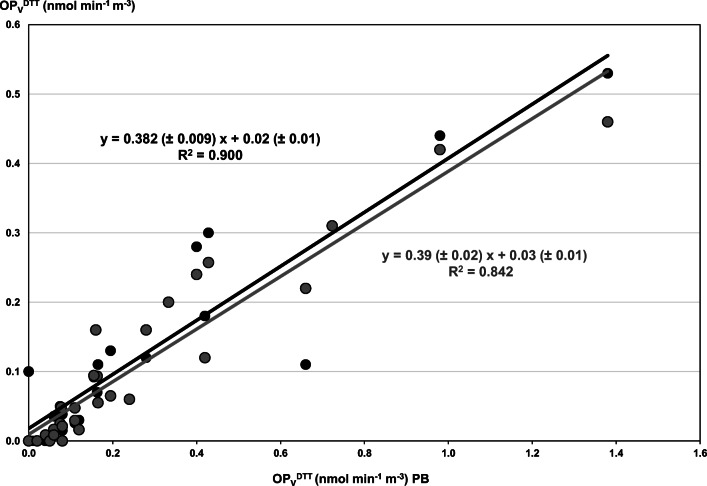
Correlation between OP_V_^DTT^ responses of all the investigated PM_2.5_ samples after different extraction procedures: linear relationships between Gamble (light grey points and line) and MeOH (black points and line) compared with PB extractions. Insets: equations of the best fitting straight lines

Overall, all the OP_V_^DTT^ responses obtained with the different solvents resulted significantly inter-correlated (Pearson coefficient at *p* ≤ 0.01, Table 2), and OP_V_^DTT^ with Gamble and MeOH extraction showed linear relationships (*R*^2^ ≥ 0.84) with that with PB (Fig. 2). This highlights that the various extraction solvents have similar effects on the DTT reactivity for all the samples, and thus they all may be likely used for OP^DTT^ assessment. However, the data plotted in Fig. 2 clearly show that the OP_V_^DTT^ responses after PB extraction were nearly double and with larger variation range than the others, so that the slope values of the best fitting straight lines are close to 0.4. Therefore, we can conclude that, among the investigated solvents, the phosphate buffer is the best solvent to choose, as it provides the highest extraction efficiency and thus the most sensible measures.

Otherwise, OP_V_^AA^ responses showed weaker correlations among the data with different solvents, with no significant correlation between Gamble and MeOH extraction for the whole data set (Table 2) and the URB samples (Table S4). This suggests that the investigated solvents yielded different extraction recovery of the components mostly effective towards the AA assay, that are mainly vehicle metals from traffic source (Calas et al. 2018; Charrier and Anastasio 2015; Crobeddu et al. 2020; Fang et al. 2016; Janssen et al.^2015^; Pietrogrande et al. 2019a; Simonetti et al. 2018; Velali et al. 2016). This is consistent with the finding that the OP_V_^AA^ responses with the different solvents were significant inter-correlated for the RUR samples (*p* <0.01, Table S5), where the contribution of redox-active metals is less dominant.

Furthermore, in order to investigate the specific role of the extraction procedure on each OP assay, the intercorrelation between OP_V_^DTT^ and OP_V_^AA^ responses was explored for each extraction procedure. No significant correlation was found between the responses of the two assays in all the data set (Table 2) and by separately investigating URB and RUR samples (Tables S4 and S5). This confirms the finding that the two OP assays display different sensitivity towards the same redox-active species present in PM, because they capture the redox reactions of different species (Bates et al. 2019; Calas et al. 2018; Charrier and Anastasio 2015; Fang et al. 2016; Janssen et al. 2015; Jiang et al. 2019; Simonetti et al. 2018), although also contrasting results are reported in literature on the topic (Bates et al. 2019; Pietrogrande et al. 2019a; Visentin et al. 2016).

### OP^DTT^ and OP^AA^ responses of individual redox-active species

The study was extended to laboratory solutions of individual redox active species, in order to support explanation of the obtained results with experimental data and integrate hypotheses derived from the literature (Bein and Wexler 2015; Roper et al. 2019; Yang et al. 2014). The rate of DTT and AA loss was assessed for both standard solutions and spiked blank filters in the three extracting conditions, also extending to ultrapure Milli-Q water, as a control solvent. Among the four tested compounds, two were quinones, namely 9,10-phenantrenequinone (9,10-PNQ), that has been found by far the most reactive quinone to DTT assay, followed by 1,2-naphthoquinone (1,2-NPQ). Others were two metals, showing high activity in AA oxidation, mainly the most reactive Cu, followed by Fe, that is the most abundant metal in PM (Calas et al. 2018; Charrier and Anastasio 2015; Simonetti et al. 2018; Tuet et al. 2017; Verma et al. 2015). Different concentrations of standard compounds were tested to represent the range of atmospheric concentrations and obtain comparable OP responses with those from real PM samples (Pietrogrande et al. 2019a; Visentin et al. 2016). The results obtained for each individual species (mean values ± SD, *n* ≥ 3) are reported in Table 3.

**Table 3:** OP^DTT^ and OP^AA^ responses (depletion rate nmol min^−1^) of standard solutions of redox-active species. OP assays were performed in pure water, phosphate buffer and Gamble’s solution media on the laboratory solutions and after extraction with different solvents on spiked blank quartz filters. For each species, measurements were repeated at least 3 times (mean and standard deviation, *n* ≥ 3)

**OP**^**DTT**^ (nmol min^-1^)				**OP**^**AA**^ (nmol min^-1^)			
Standard solution	Pure water	Phosphate buffer	Gamble’s solution	Standard solution	Pure water	Phosphate buffer	Gamble’s solution
Cu^2+^ (1 μM)	3.49±0.12	3.33±0.19	2.49±0.14*	Cu^2+^ (0.17 μM)	5.42±0.24	5.31±0.31	4.49±0.27
Fe^2+^ (1 μM)	0.52±0.03	0.48±0.03	0.34±0.03	Fe^2+^ (1 μM)	1.12±0.06	1.07±0.06	0.97±0.06
1,2-NPQ (0.5 μM)	1.92±0.09	1.82±0.11	1.13±0.07*	1,2-NPQ (0.5 μM)	4.95±0.25	4.81±0.28	3.91±0.21
9,10-PNQ (0.17 μM)	1.42±0.06	1.38±0.08	1.26±0.07	9,10-PNQ (1 μM)	0.91±0.05	0.91±0.06	0.60±0.03*
**OP**^**DTT**^ (nmol min^-1^)							
Spiked blank filters	Pure water	Phosphate buffer	Gamble’s solution	Methanol			
Cu^2+^ (0.5 μM)	1.93±0.10	1.84±0.11	1.21±0.09*	0.23±0.02*			
Fe^2+^ (1 μM)	0.58±0.04	0.55±0.04	0.38±0.03	ND			
1,2-NPQ (0.5 μM)	1.20±0.07	1.17±0.08	0.75±0.05*	1.41±0.11*			
9,10-PNQ (0.17μM)	1.05±0.08	1.06±0.09	0.95±0.04	1.65±0.12*			
**OP**^**AA**^ (nmol min^-1^)							
Spiked blank filters	Pure water	Phosphate buffer	Gamble’s solution	Methanol			
Cu^2+^ (0.5 μM)	4.02±0.25	4.76±0.28	4.35±0.26	ND			
Fe^2+^ (1 μM)	1.25±0.08	1.29±0.09	1.12±0.06	ND			
1,2-NPQ (0.5 μM)	3.52±0.19	3.62±0.23	2.95±0.15*	4.87±0.38*			
9,10-PNQ (0.17μM)	0.85±0.03	0.78±0.05*	0.53±0.01	1.38±0.08*			

*ND* OP response lower than that of the blank quartz filter

First, the effect of the medium assay was investigated for the three aqueous solutions on each individual standard by measuring OP^DTT^ and OP^AA^ responses. Overall, similar results were obtained suggesting that the choice of the aqueous solution had no significant effect on the DTT and AA assay reactivity. We only observed a significant effect (*p* < 0.05, indicated by asterisk in the Table 3) for the Gamble’s solution, with a lower OP^DTT^ for Cu^2+^ and 1,2-NPQ and a lower OP^AA^ for 9,10-PNQ. Consistent with our results, the detailed investigation of Calas (Calas et al. 2017) ascribed a similar OP^DTT^ trend to the presence in G of complexing anions (orthophosphates, carbonates, acetates) and functional groups (carboxyl from glycine, citrate, and amines from glycine), that may chelate metals and thus reduce their availability to redox reactions. They also found a similar G decreasing effect on quinones, that was unexpected, as organic compounds do not form strong complexes with chelating species. As likely explanation, they suggested that the Gamble medium may be less favorable to electron transfer than simpler aqueous solutions and/or that quinones may be transformed during the extraction step and storage. The trend of OP^AA^ responses, with lower values in G than in PB, is consistent with the previous Authors’ results, showing that OP^AA^ values decreased by adding further components to the phosphate buffer (Pietrogrande et al. 2019b).

### Explanation of effects of extracting solutions on OP_V_^DTT^ and OP_V_^AA^ responses

#### Effect of the extracting solutions: comparison among aqueous solutions

Then, the role of the three aqueous solutions was investigated by extracting blank filters spiked with laboratory standards, in order to explain the specific effect of each extraction procedure on metals and quinones. The obtained OP^DTT^ and OP^AA^ values confirmed the above described trend, with nearly the same OP responses measured with pure water and phosphate buffer, that were higher than those with Gamble’s solution*.* Indeed, we observed a significant difference (*p* < 0.05, indicated by asterisk in the Table 3) only for OP^DTT^ of Cu^2+^ and 1,2-NPQ and for OP^AA^ of 1,2-NPQ and 9,10-PNQ. This may be ascribed to the dominant role of chelating agents in G medium in inhibiting DTT and AA depletion rate (Calas et al. 2017; Pietrogrande et al. 2019b). Otherwise, it must be underlined that such G complexing components can also display an opposite effect of increasing OP responses, as they are able to extract larger fractions of metal content from the filter, according to their chelating strengths (Collins et al. 2015; Leclercq et al. 2017; Luo et al. 2019; Mukhtar et al. 2015; Wiseman, 2015). Therefore, the overall variation may be ascribed to the combination of two contrasting contributions, namely solvent extraction efficiency to retrieve redox-active components into solution and reactivity of individual species in the two assay media. This may be likely the reason why the OP_V_^AA^ variations for Cu and Fe are weaker than those of OP_V_^DTT^ (G nearly 67% of PB), as the AA assay is more sensitive to the possible larger metal content extracted by the Gamble’s solution.

#### Effect of the extracting solutions: methanol vs. aqueous solutions

Finally, the effect of the aqueous extraction media was compared to that of methanol by measuring OP^DTT^ and OP^AA^ of blank filters spiked with laboratory standards, after extraction with the four solvents. Our experimental results clearly show that, compared with the used aqueous solutions, methanol extraction yielded significantly higher (at *p* < 0.05, asterisk in Table 3) DTT and AA responses for quinones and lower for Cu and Fe. Such a trend may be ascribed to variation in the extraction efficiency of the solvents, consistent with the decreased solubility of metal ions in MeOH compared with water solutions (Roper et al. 2019; Wei et al. 2019; Xing et al. 2019). Furthermore, MeOH has been found more effective than water to retrieve DTT reactive organic components, mainly oxidized organics (Bein and Wexler 2015; Gao et al. 2017; Janssen et al. 2015; Roper et al. 2017; Roper et al. 2019; Verma et al. 2015; Yang et al. 2014).

Overall, in our study, the OP_V_^DTT^ and OP_V_^AA^ values of MeOH extracts of real samples were lower than those of PB extracts and like those of G extracts, with magnified differences for the urban samples. This pattern resembles that of metals, suggesting that the oxidative properties of our real samples are mainly driven by transition metals, among the redox-active species. Such a conclusion is also consistent with the finding that all the samples showed higher responses of the AA assay, that is more sensible to metals than the DTT assay (Bates et al. 2019; Calas et al. 2018; Fang et al. 2016; Janssen et al. 2015; Pietrogrande et al. 2019a; Simonetti et al. 2018; Visentin et al. 2016). The predominant role of metals, mainly more abundant iron, in the PM oxidative properties is consistent with several literature data (Charrier and Anastasio 2015; Crobeddu et al. 2020; Perrone al. 2019; Pietrogrande et al. 2019a; Simonetti et al. 2018; Velali et al. 2016; Wei et al. 2019). However, caution must be exercised when interpreting such results, since the current study dataset lacked the Cu concentration, that is one of the most sensitive metals driving OP responses, mainly those of the AA assay.

### Correlation between OP responses in different extraction solutions and PM chemical constituents

In addition to OP responses, PM_2.5_ mass concentrations and selected chemical components were measured for each PM_2.5_ sample, i.e., organic and elemental carbon, secondary ions, and some soluble transition metals (Tables S1 and S2 in Supplementary material). From the individual data, the mean and SD values were computed for all the samples and for urban and rural filters, separately (Table 1). The Student’s *t* test was applied to single out significant (*p* < 0.05) differences between sites (indicated by **†** in Table 1). Among the investigated parameters, higher values were measured at the URB than at RUR site for PM_2.5_ mass (16.8 ± 5.2 μg m^−3^ vs. 13.8 ± 3.7 μg m^−3^), EC (1.04 ± 0.43 μg m^−3^ vs. 0.54 ± 0.17 μg m^−3^), total metals (166 ± 82 ng m^−3^ vs. 26.6 ± 16.1 ng m^−3^), and iron (128 ± 58 ng m^−3^ vs. 33.6 ± 27 ng m^−3^), as indicated by **†** in Table 1. This is consistent with a higher impact from anthropogenic source emissions at the urban site, mainly related to traffic, as previously found by the Authors at the same sites (Pietrogrande et al. 2016; Pietrogrande et al. 2019a, b).

The correlation analysis was performed to associate OP_V_ response with PM_2.5_ chemical composition with the main aim to highlight if the different extraction conditions may vary such associations. Overall, the obtained results showed similar behavior for the three investigated solvents, suggesting no major impact of the extraction solvent on these correlations (Table 2). In general, OP_V_^DTT^ data resulted more widely correlated with several PM components, including PM_2.5_ mass concentration, compared with OP_V_^AA^. Consistently with other papers, DTT assay mainly responded to the organic compounds, traced by OC and EC (*p* < 0.01), that represent fuel vehicular (EC) and biomass burning emissions (Levoglucosan and K, *p* < 0.05), and also to secondary atmospheric processes, traced by NH_4_^+^, NO_3_^−^ and SO_4_^2−^ ions (*p* < 0.05). In addition, OP_V_^DTT^ responses resulted correlated with traffic-related metals, mainly non-exhaust traffic emissions, such as Fe, Mn, Zn, and Pb (*p* < 0.01). By separating URB and RUR samples, we can observe that the OP_V_^DTT^ values of the URB site are more strongly associated with metals, while those at the RUR site with OC and EC (Tables S4 and S5). This is consistent with the predominant role of secondary organic carbon, OC, and biomass burning at the rural site, as it was less impacted by traffic emission (metals) (Pietrogrande et al. 2019a). Among the extraction solvents, Gamble’s solution generated OP_V_^DTT^ values better correlated with metal concentration, that is consistent with its higher extraction power towards these inorganic components.

Concerning OP_V_^AA^ responses, significant associations (*p* < 0.01) were found only with EC after PB and GS extraction and none with other investigated parameters (Table 2), independent of the extraction procedure, neither grouping URB nor RUR samples (Tables S4 and S5). Such results are consistent with the previously discussed lack of intercorrelation between OP_V_^DTT^ and OP_V_^AA^ responses, as they are differently correlated with the same redox-active PM components (Bates et al. 2019; Calas et al. 2018; Fang et al. 2016; Janssen et al. 2015; Pietrogrande et al. 2019a; Simonetti et al. 2018; Visentin et al. 2016).

Based on these results, we can confirm that oxidative properties of the investigated PM_2.5_ samples depend on both transition metals and organics, with a stronger association of DTT reactivity with chemical components. It should, however, be noted that caution must be exercised when interpreting correlation results, as some conclusions may be potentially affected by other PM components not identified in this study, that may induce ROS production. Further, it is difficult to identify the relative contribution of each component, as it is given by the combination of its individual reactivity associated with its concentration level in PM.

## Conclusions

Overall, the obtained results highlighted that the investigated filter extraction procedures generated differences in the measured oxidative potential of the PM_2.5_ samples. For our current research goals, of the three tested solvents, the phosphate buffer resulted the solvent of choice, since it provided the most sensible measure of OP^DTT^. Although transition metals and quinones have been identified as the chemical components mainly responsible of such results, mechanisms driving the solvent extraction effects on OP responses must be interpreted with caution, as several redox active PM components are involved in ROS production and synergic/antagonistic interactions may be likely operating.

This research used PM_2.5_ filters from an urban, traffic-dominated, and rural locations collected during spring. To generalize these findings, we plan to explore this topic in the future by looking at OP_V_^DTT^ and OP_V_^AA^ responses of PM_2.5_ with different source contributions and with a more detailed chemical characterization.

This research emphasizes the importance for considering each step of the OP assay procedure in order to select a standardized protocol to enable accurate interlaboratory comparable OP responses to be included in toxicology research on exposures to ambient PM_2.5_ mixtures.

## Supplementary Information

ESM 1 (DOCX 35 kb)

Further information regarding the measured values and correlations among the data are shown in the supporting information file. This file also includes further correlations among the data.

## Data Availability

The datasets used and analyzed during the current study are available from the corresponding author on reasonable request.
